# Risk factors and management of biliary leakage after Endocystectomy for hepatic cystic echinococcosis

**DOI:** 10.1371/journal.pntd.0011724

**Published:** 2023-10-31

**Authors:** Sepehr Abbasi Dezfouli, Ahmad El Rafidi, Ehsan Aminizadeh, Ali Ramouz, Mohammed Al-Saeedi, Elias Khajeh, Markus Mieth, Tim Frederik Weber, De-Hua Chang, Kathrin Hoffmann, Markus W. Büchler, Arianeb Mehrabi

**Affiliations:** 1 Department of General, Visceral, and Transplantation Surgery, Heidelberg University Hospital, Heidelberg, Germany; 2 Liver Cancer Center Heidelberg (LCCH), Heidelberg University Hospital, Heidelberg, Germany; 3 Department of Diagnostic and Interventional Radiology, Heidelberg University Hospital, Heidelberg, Germany; Ministerio de Salud Publica, ARGENTINA

## Abstract

**Background:**

Endocystectomy is a conservative surgical approach to managing cystic echinococcosis. Bile leakage is the main complication of this technique. The aim of this study was to evaluate the factors associated with bile leakage and to assess the outcomes and cost efficiency of strategies used to treat bile leakage.

**Methodology/Principal findings:**

Patients who underwent endocystectomy between 2005 and 2020 were included. The preoperative characteristics, intra- and postoperative outcomes, hospital costs, and cost efficiency (the Diagnosis-Related Group reimbursement minus the overall cost) were evaluated prospectively. A total of eighty patients with 142 cysts were included. Postoperative complications occurred in 17 patients (21%), including 11 patients with bile leakage (type A: 1, type B: 6 and type C: 4 patients, total 13%). Bile leakage was more frequent in patients with preoperative MRI signs of cysto-biliary fistulas or intraoperative visible cysto-biliary fistulas (*p* = 0.03 and *p* = 0.04, respectively) and in patients with cysts larger than 8 cm (*p* = 0.03). Patients with bile leakage who underwent reoperation (type C) had significantly shorter hospital stays (9 vs. 16 days, *p*<0.01) and better cost efficiency than those who received radiologic or endocscopic interventions (€2,072 vs. -€2,097 *p* = 0.01). No mortality was observed, and recurrence was seen in two patients.

**Conclusions/Significance:**

Endocystectomy is a safe and efficient technique. Preoperative and intraoperative cysto-biliary fistulas and a cyst diameter larger than 8 cm are correlated to postoperative bile leakage. Early operative management of bile leakage reduces hospital stay and improves cost efficiency compared with radiologic or endoscopic treatments.

## Author summary

Endocystectomy is a conservative surgical approach to managing hepatic cystic echinococcosis (CE). Bile leakage is the major complication of this technique. In the current study, we evaluated the patients who underwent endocystectomy and assessed the factors associated with bile leakage. The operative and postoperative outcomes and cost efficiency of strategies, which was used to treat bile leakage were also evaluated. Eighty patients with 142 cysts were included. Among them eleven patients shown bile leakage. Four patients underwent reoperation to treat bile leakage, while six patients received radiologic intervention. We found that cysto-biliary fistula and cyst size >8 cm correlate with bile leakage after endocystectomy. We also concluded that early operative management of bile leakage reduces hospital stay and improves cost efficiency compared with radiologic or endoscopic treatments.

## Introduction

Cystic echinococcosis (CE) is a complex parasitic disease caused by Echinococcus granulosus with a low incidence in Europa [1]. The organs most likely to be infected are the liver and lungs [2]. Hepatic CE is largely asymptomatic, but can cause complications due to compression of neighboring organs and the biliary tree [3].

Treatment options for CE include the watch-and-wait approach, medical treatment (with benzimidazoles), percutaneous interventions, and surgery [3–5]. The treatment strategy and indications for surgical treatment are decided based on the World Health Organization (WHO) CE cyst classification [3]. Based on these recommendations, surgery is the treatment of choice for active large CE cysts that are complicated (such as cases with cysto-biliary fistulas, ruptured cysts, or organ compression) or cannot be treated by medication, the percutaneous approach, or the watch-and-wait strategy. The ideal surgical method is still a matter of debate. However, new studies have shown some advantages of endocytectomy as a conservative surgery [6].

The main complication of endocystectomy is bile leakage, which has been reported in up to 20% of patients in large studies [6,7]. Bile leakage is difficult to manage and can lead to prolonged hospital stay and higher admission costs [7,8]. Limited data are available on the factors related to postoperative bile leakage, and even less data is available on the management of bile leakage after endocystectomy [7,9]. In this study, we report the outcomes of patients who underwent endocystectomy in our center, focusing on the factors that influence bile leakage. We also evaluated the outcomes and cost efficiency of two treatment strategies for postoperative bile leakage (interventional management and early reoperation).

## Methods

### Ethics statement

The study protocol was approved by the regional ethics committee of the medical faculty of Heidelberg University (approval number: S-754/2018) and was conducted in accordance with good clinical practice guidelines and the declaration of Helsinki.

### Study design

The data of patients who underwent endocystectomy for liver CE between 2005 and 2020 were investigated from a prospectively maintained database. We included patients who underwent elective endocystectomy and excluded patients who received emergency surgery after cyst rupture or radical liver resection. Signed consent was obtained from all patients before using their clinical data.

### Surgery indications and patient cohort

Diagnoses and surgical indications were given by the interdisciplinary team at the Heidelberg Echinococcosis Treatment Center and were based on WHO recommendations. The center comprises the Clinical Tropical Medicine Unit, Department of Radiology, Department of General Surgery, Department of Thoracic Surgery, Interdisciplinary Center for Endoscopy, and the Department of Parasitology. Diagnosis was based on imaging, ultrasound, and/or magnetic resonance imaging (MRI) and, eventually, on serology. Surgery was indicated in patients with 1) WHO cyst stages CE 1, 2, and 3a liver cysts of > 10 in diameter; 2) CE 3b cysts, independent of size; and 3) CE1 to CE3b cysts that had been unsuccessfully treated with other modalities [3].

### Surgical procedure

Patients scheduled for endocystectomy received preoperative medical therapy with albendazole two days before surgery, and this treatment was continued for at least four weeks after surgery, to reduce the chances of the preoperative cyst viability as well as the postoperative cyst recurrence [10]. Our standardized endocystectomy technique was described in an earlier report [11].

In all Patients a laparotomy is performed using a midline incision with a right lateral extension if necessary. After the mobilization of the liver, we use intraoperative ultrasound examination to locate the cysts. To protect the surrounding tissues from cyst content, we use two layers of sponges (the first layer is moistened with 0.9% normal saline and the second layer with 20% sodium chloride). Cyst content is removed using a 12-mm trocar under permanent suction. Afterwards, the capsule is partially resected and the residual cavity is explored for cysto-biliary communications. Visible cysto-biliary communications are closed with single stitches. Finally, the edge of the cyst is sewn over to prevent bile leakage. If possible, an omentoplasty is performed. A cholecystectomy is not performed routinely. Also, bile leak tests such cholangiogram or White test are not performed routinely, but in some cases at surgeon discretion. Since bile leakage is the main problem after endocystectomy, a surgical drain (open passive drain connected to a stoma bag) is placed as standard.

### Patient data collection and measurements

#### Preoperative evaluations

Patient demographic and clinical data were recorded, including gender, age, the American Society of Anesthesiologists (ASA) classification, number of cysts, diameter of cysts, location of cysts, and the WHO classification of the cysts. The preoperative diagnosis of cysto-biliary fistulas was given in patients using MR imaging. In these patients, the presence of hypointense structures within the bile ducts, i.e. migrated hydatid debris were interpreted as indirect signs for the presence of cysto-biliary fistulas, and cyst wall defects with a continuity between the cyst cavity and the biliary tree or the adjacent organs, e.g. intestinal tract were interpreted as direct signs of cysto-biliary fistulas. Dilated bile ducts alone were not regarded as a reliable sign for the presence of cysto- biliary fistulas.

#### Intraoperative evaluations

Intraoperative blood loss was measured by adding the blood absorbed by gauzes and the amount of blood collected in the suction bottle. The amount of transfused blood products including red blood cells (RBC) and fresh frozen plasma (FFP) was recorded. Operation duration was defined as the time from the start of the skin incision to the end of the surgical procedure.

#### Postoperative data collection

Drain fluid was routinely analyzed for bilirubin and the drains were removed after the third postoperative day if no bile leakage was detected. Bile leakage was assessed according to the definition of the International Study Group of Liver Surgery (ISGLS) [12]. No routine radiological diagnostic was performed after this point. After removing the surgical drains, bile leakage was diagnosed in patients with symptoms such as fever, or in asymptomatic patients with increased infection parameters and a CT-scan was performed, and if a collection was seen, an interventional drain was inserted. Collected fluid was analyzed for bilirubin to confirm the diagnosis. If a biliary leakage was seen in these drains, a daily monitor of the draining volume and biliary concentration was performed. If no spontaneous healing tendency was seen, an Endoscopic retrograde cholangiopancreatography (ERCP) was performed with sphincterotomy and a stent is placed in the common bile duct to facilitate the bile flow. If the patients were symptomatic, empirical antibiotic therapy based on surgeons’ choice (Ampicillin/Sulbactam, Piperacillin/Tazobactam or Ceftriaxone + Metronidazole) were prescribed. Other surgical complications were also recorded and classified based on the Clavien–Dindo classification. The duration of intensive care unit (ICU) stay, intermediate care unit (IMC) stay, and hospital stay as well as the 90-day mortality were recorded. After discharge from hospital, patients were followed up in the outpatient clinic of the surgical department and in the special clinic for CE in the tropical medicine unit, which carries out long-term follow-up for recurrence and mortality. All patients were followed up after operation every month for three months, then every three months for duration of one year, and then every six months [11,13]. To detect recurrences, an ultrasound or MRI was performed within one month of the operation and once per year afterwards [11].

### Management of bile leakage

Based on our policy, patients with early postoperative bile leakage detected within the first three postoperative days undergo an immediate re-operation. Patients with bile leakage detected after the third postoperative day undergo an endoscopic retrograde cholangiography (ERC) with sphincterotomy and a stent is placed in the common bile duct to facilitate bile flow. Surgical drains were left in place until bile leakage stopped. If the patient’s condition did not improve or signs of infection and fever were not resolved, a CT scan was performed. If undrained collections were detected, an additional interventional drain was inserted.

### Evaluations of hospital costs and cost efficiency

#### Diagnosis-related reimbursements

These were calculated based on the Diagnosis-Related Group (DRG) using the German procedure coding system (OPS) [14]. These reimbursements represent the total amount reimbursed by the payer for care during the index hospital admission and the surgical-related costs.

#### Total hospital costs

These costs were based on our finance department’s database. They covered all treatment costs, including surgical and interventional costs, the costs of ICU/IMC stay and ward stay, medications, resources, and staff.

#### Cost efficiency

This was calculated as the difference between DRG reimbursements and total hospital costs.

### Statistical analysis

Outcomes were extracted directly or calculated from the raw data. Continuous variables were presented as median and interquartile range (IQR), while categorical variables were presented as frequency distributions (proportions and percentages). The cut-off values of cyst size were calculated using the receiver operating characteristic (ROC) curve based on bile leakage. The area under the ROC curve (AUROC) was evaluated using 95% confidence intervals (CIs). Discrimination was categorized as acceptable if the AUROC was above 0.6 and as excellent if it was above 0.7. Continuous data were analyzed using the Student’s t-test or the Mann–Whitney U test and categorical data were analyzed using the chi-square or Fisher’s exact test. A two-sided *p* value of <0.05 was considered significant. SPSS software (Version 27, SPSS Inc., Chicago, IL, USA) was used for data analysis.

## Results

### Preoperative data

Eighty patients with 142 cysts underwent endocystectomy between 2005 and 2020. Of these, 40 patients (50%) were female. The mean ± SD age of included patients was 32 ± 12.5 years. Most patients were categorized as ASA II (72.5%) or ASA I (22.5%). Single cysts were reported in 44 patients (55%). The mean number of cysts per patient was 1.7 ± 1.3, ranging from one to eight cysts. The mean cyst diameter was 7.4 ± 3.6 cm and 97/142 cysts (68%) were located in the right lobe of the liver and 45/142 cysts (32%) were located in left lobe of the liver (Table 1). The WHO classification of the operated cysts was as followed: CE1 25 cysts, CE2 45 cysts, CE3a 26 cysts, CE3b 34 cysts. CE4 and CE5 cysts 12 (these cysts were resected because of their proximity to other cysts and were not the primary indication for the operation).

**Table 1:** Demographic, intraoperative, and postoperative data of patients with endocystectomy.

Parameters		Total n = 80 patients with 142 cysts
Gender, n (%)	Male	40 (50)
	Female	40 (50)
Age (yr.), Median (IQR)		31 (23–39.5)
ASA classification n (%)	ASA 1	18 (22.5)
	ASA 2	58 (72.5)
	ASA 3	4 (5)
Number of cysts	Single	44 (55)
	Multiple	36 (45)
Location of the cyst, n (%)	Right lobe	97 (68)
	Left lobe	45 (32)
Blood loss (ml), Median (IQR)		100 (50–200)
Intraoperative RBC transfusion, n (%)		2 patients (2.5), 3 units
Intraoperative FFP transfusion, n (%)		2 patients (2.5), 6 units
Operation duration (min), Median (IQR)		172 (150–220)
Complication, n (%)		17 (21.2)
Bile leakage, n (%)	Bile leakage type A	1 (1.2)
	Bile leakage type B	6 (7.5)
	Bile leakage type C	4 (5)
Other Complications, n (%)	Fluid collection	2 (2.5)
	Pancreas fistula	1 (1.25)
	Ulcer perforation	1 (1.25)
	Pleural effusion	1 (1.25)
	Liver abscess	1 (1.25)
ICU/IMC stay (days)*, Median (IQR)		2 (1–4)
Hospital stay (days), Median (IQR)		10 (8–13)
Recurrence, n (%)		2 (2.5)
Mortality, n (%)		0 (0)
DRG Reimbursement (€), Median (IQR)		8702 (7486–13974)

Abbreviations: n: number; IQR: interquartile range; ASA: American Society of Anesthesiologists; RBC: red blood cell, FFP: fresh frozen plasma; yr: years; cm: centimeters; ml: milliliters; min: minutes; ICU: intensive care unit; IMC intermediate care unit

*: The median is calculated in patients, who had ICU/IMC stay, not all the patients.

### Intraoperative data

The mean intraoperative blood loss was 206 ± 38 ml; RBC were transfused in two patients (2.5%, three units) and FFP was transfused in two patients (2.5%, six units). The mean operation time was 188 ± 65 minutes. No intraoperative complications occurred (Table 1). In 33 Patients, a cysto-biliary was observed intraoperatively.

### Postoperative data

Postoperative complications were seen in 17 patients (21%), six of which (7%) were classified as Clavien–Dindo grade ≥IIIb. Eleven patients developed bile leakage (14%). This was diagnosed based on increased bilirubin in the intraoperative drains in nine cases (82%) and in the two remaining cases, bile leakage occurred after the drains were removed. Type B bile leakage was seen in six patients (7%), which underwent radiologic or endoscopic interventions and type C bile leakage was seen in four patients (5%), who underwent reoperation. Patients with type C bile leakage underwent immediate reoperation during the first three postoperative days. In all these patients, bile leaked from a cysto-biliary fistula at the endocystectomy site was seen. No complications after the reoperation were seen in these patients.

Six patients showed other complications. One patient who underwent endocystectomy and splenectomy for CE in the liver and spleen developed a pancreatic fistula, which was treated interventionally. Two patients developed perihepatic fluid collections and one patient developed a pleural effusion. One patient developed a duodenal ulcer perforation and was reoperated. The remaining patient developed bile duct necrosis and a liver abscess, which was treated with a left-sided hemihepatectomy.

Thirteen patients (16%) were admitted to the ICU/IMC after surgery (median stay: 1 (1–4.5) days). The median hospital stay of all patients was 10 (8–13) days. No 90-day mortality was detected. The median duration of follow-up after endocystectomy was 26 months (range: 3–179 months), during which CE recurrence was reported in two patients (2.5%)–one 15 months after surgery and close to the endocystectomy site, and the other 23 months after surgery and in the endocystectomy site. No patients died during follow up (Table 1).

### Subgroup analysis of patients with complications

There were no differences (*p*>0.05) in age, gender, ASA classification, and number of cysts between patients with complications and patients without complications (Table 2). Also, there was no difference in WHO classification of the cysts between two groups. In patients with complications, the cyst diameter was significantly larger (8.5 (6.1–12) cm vs. 6.5 (4.45–9.65) cm, *p* = 0.031) and significantly more patients had cysts larger than 8 cm (65% vs. 35%, *p* = 0.03). Postoperatively, patients with complications had significantly longer ICU/IMC stays (0 with IQR of (0–11) vs. 0, *p* = 0.008) and hospital stays (16 (13–30) vs. 9 (7–11) days, *p*<0.001) than those without complications.

**Table 2:** Comparison of patient characteristics, intraoperative outcomes, and postoperative outcomes of A) patients with and without complications, B) patients with and without bile leakage, and C) patients with type B and type C bile leakage.

Parameters	A			B			C		
	With complications (n = 17)	Without complications (n = 63)	P value	With bile leakage (n = 11)	Without bile leakage (n = 69)	P value	Bile leakage B (n = 6)	Bile leakage C (n = 4)	P value
Number of cysts, Median (IQR)	2 (1–2.5)	1 (1–2)	0.231	1 (1–2)	1 (1–2)	0.780	1.5 (1–2)	1 (1–2.5)	0.762
Diameter of cysts (cm), Median (IQR)	8.5 (6.1–12)	6.5 (4.45–9.65)	**0.031**	8.5 (7–11)	6.8 (4.5–10)	**0.034**	10.5 (5.9–18.5)	6.95 (3.97–8.35)	0.171
Diameter of cysts >8 cm, n (%)	11 (64.7)	23 (36.5)	**0.037**	8 (72.7)	26 (37.7)	**0.047**	5 (83.3)	2 (50)	0.261
Blood loss (ml), Median (IQR)	150 (50–375)	100 (50–200)	0.224	150 (50–200)	100 (50–200)	0.415	150 (50–237)	125 (62–187)	0.762
Operation duration (min), Median (IQR)	180 (146–230)	167 (150–212)	0.637	155 (130–195)	172 (150–224)	0.231	168 (128–203)	146 (126–1569)	0.352
ICU/IMC stay (days), Median (Range)	0 (0–11)	0 (0–2)	**0.008**	0 (0–3)	0 (0–11)	0.517	0 (0–3)	0	0.114
Hospital stay (days), Median (IQR)	16 (13–30)	9 (7–11)	**<0.001**	16 (13–23)	9 (7.75–12)	**0.001**	21.5 (14.7–29.7)	14 (7.7–15.7)	**0.038**

Abbreviations: n: number; IQR: interquartile range; ml: milliliters; min: minutes; ICU: intensive care unit; IMC intermediate care unit; yr: year; cm: centimeter

### Subgroup analysis of patients with bile leakage

There were no significant differences in age, gender, ASA class, number of cysts, and operative parameters such as blood loss and operation time, and ICU/IMC between patients with bile leakage and patients without bile leakage (Table 2). Patients with bile leakage had significantly longer hospital stays (16 (13–23) vs. 9 (7.75–12) days, *p* = 0.001) than those without bile leakage. In patients with bile leakage, the cyst diameter was significantly larger (8.5 (7–11) cm vs. 6.8 (4.5–10) cm, *p* = 0.034) and significantly more patients had cysts larger than 8 cm (73% vs. 38%, p = 0.04). In patients with bile leakage, blood loss, operation time, ICU/IMC stay, and hospital stay were similar between patients with type B and C bile leakage. The number and diameter of cysts also did not differ between patients with type B and C bile leakage. Patients with type B bile leakage had a significantly longer hospital stay than patients with bile leakage type C (21.5 (14.7–29.7) days vs. 14 (7.7–15.7) days, *p* = 0.038).

### Cysto-biliary fistula and bile leakage

Cysto-biliary fistulas were diagnosed radiologically before surgery in 21 patients (26%) from which 18 patients showed cysto-biliary fistulas intraoperatively (specificity: 94%). Thirty-three patients showed cysto-biliary communication during surgery, but 15 of these did not show radiologic signs of fistula during the preoperative diagnostics (sensitivity: 54%). Significantly more patients with a preoperative diagnosis of cysto-biliary fistula had bile leakage after endocystectomy than patients without a preoperative diagnosis did (*p* = 0.032). Furthermore, the incidence of bile leakage was significantly higher in patients with intraoperative cysto-biliary fistula than in patients without intraoperative fistula (*p* = 0.044) (Table 3).

**Table 3:** Relation of cysto-biliary fistula and postoperative bile leakage.

		Intraoperative visible cysto-biliary fistula (n, %)		Postoperative bile leakage (n, %)		
		Yes (n = 33)	No (n = 47)	Yes (n = 11)	No (n = 69)	P value = **0.032**
Preoperative suspicion of cysto-biliary fistula (n, %)	Yes (n = 21)	18 (85.7)	3 (14.3)	6 (28.6)	15 (71.4)	
	No (n = 59)	15 (25.4)	44 (74.6)	5 (8.5)	54 (91.5)	
Postoperative bile leakage (n, %)	Yes (n = 11)	8 (72.7)	3 (27.3)	-	-	
	No (n = 69)	25 (36.2)	44 (63.8)	-	-	
	P value = **0.044**					

### Hospital costs and cost efficiency

The mean DRG reimbursement of endocystectomy for all patients was €8,702 (€7,486-€13,974). Patients with complications had significantly higher DRG reimbursements (€14,182 (€12,364-€18,125)), than patients without complications (€8,032 (€7,430-€9,192), *p* = 0.01). Additionally, patients with bile leakage had significantly higher DRG reimbursements than patients without bile leakage (€14,182 (€12,317-€17,405) vs. €8,243 (€7,486 -€12,787), *p*<0.001).

Although not statistically significant, DRG reimbursements of patients with bile leakage type C were higher than reimbursements of patients with bile leakage type B (€20,851 (€12,879-€27,961) vs. €14,078 (€11839-€15587), *p* = 0.352). The overall costs for patients with bile leakage type B was higher than those for patients with bile leakage type C, although this difference was not statistically significant (€16,661 (€10,436-€20,317) vs. €12,881 (€11,597-€22,161), *p* = 0.914). However, the difference between overall cost and DRG reimbursement (cost efficiency) between patients with bile leakage type B and C showed a significant difference (€-2,097 (€-6,290- €-262) vs. €2,072 (€891-€12,088), *p* = 0.010) (Table 4).

**Table 4:** Comparison of cost efficiency outcomes of patients with bile leakage type B and type C.

Parameters	Bile leakage B (n = 6)	Bile leakage C (n = 4)	P value
DRG reimbursement (€), Median (IQR)	14078 (11839–15587)	20851 (12879–27961)	0.352
Overall cost (€), Median (IQR)	16661 (10436–20317)	12881 (11597–22161)	0.914
cost efficiency (DRG reimbursement—overall cost) (€), Median (IQR)	-2097 (-6290 to -262)	2072 (891–12088)	**0.010**

Abbreviations; IQR: interquartile range; DRG: Diagnosis-Related Group; n: number

## Discussion

In the current study on 80 patients undergoing endocystectomy, we observed 21% morbidity, 13.7% bile leakage, and 2.5% disease recurrence. Factors associated with higher bile leakage were cyst diameters of more than 8 cm and the presence of cysto-biliary fistulas. Patients with type C bile leakage undergoing immediate reoperation showed shorter hospital stays and better cost efficiencies than those receiving conservative therapies (type B).

The decision to perform conservative surgery (endocystectomy) or radical surgery (liver resection) in patients with CE is still controversial [13]. Conservative surgery has become more agreeable among some surgeons in the last years because of its easy technique, parenchymal sparing approach, and reduced operative trauma [6,15]. Especially, it is a useful and suitable option for small centers, where hepatobiliary and pancreatic surgeons are not available, independent of the results when compared to radical treatment. On the other hand, even though some authors believe that complications such as bile leakage and recurrence are higher after endocystectomy than after radical surgery [15,16], a recent meta-analysis showed no difference in bile leakage rate between the two groups [15,17]. Another recent meta-analysis showed that endocystectomy has similar mortality and recurrence rates to radical surgeries [6]. In our study, the rate of postoperative complications was 21%, which was similar to rates reported in studies after radical surgery (17.7%) [15,17]. In our center, we performed only endocystectomy and therefore it was not possible for us to compare these two methods using our data. None of our patients died within 90 days of surgery. During a median follow-up of 26 months, two cases of relapse (2.5%) were observed, which is in line with recurrence rates of 2–15% reported in the literature [6].

The most common complication was bile leakage. We observed bile leakage in 13.7% of patients, which is similar to rates of 19–24% reported in other high-volume studies [7,18–20]. In our collective, patients had an average of two cysts and the rate of bile leakage per cyst was 8%. The length of hospital stay and cost were significantly higher in patients with bile leakage than in patients without bile leakage, which is in agreement with the findings of similar studies [21,22]. This highlights the importance of identifying predictive factors of bile leakage to indicate when preventive strategies should be implemented. We identified preoperative factors that were related to bile leakage; these were the size of the cyst and the presence of cysto-biliary fistulas. The location of the CE showed no associated with the incidence of the bile leakage. Therefore, preventive strategies could be used in patients with larger cysts and cysto-biliary fistulas, such as preoperative ERC with endoscopic sphincterotomy or Botox injection, to reduce bile leakage [23–26]. In all patients with postoperative bile leakage who underwent a reoperation, an open cysto-biliary fistula was observed as the cause of the bile leakage which was missed during the first operation. Hence, it is important not to miss these fistulas during the endocystectomy. These fistulas are easily missed intraoperatively because of the positive cyst pressure. After evacuating these cysts, the pressure is lifted, and the bile leak becomes visible. In some cases though, this leakage becomes visible not until hours after the surgery. Therefore, bile leakage could be reduced using strategies to better detect the existing cysto-biliary fistulas, such as intraoperative cholangiography, or White test [8,21,27]. These strategies are subjects to further prospective studies, which could have an important impact on reducing the postoperative bile leakage and shorten the hospital stay.

A number of large studies have reported on the results of endocystectomy for CE [7,19,28]; however, to our knowledge, none have addressed the management of postoperative bile leakage. Patient outcome is the most important consideration when choosing between different therapy strategies. In our series, patients with bile leakage in the first three days of surgery underwent an immediate reoperation. Reoperation is technically easier in the early days following the first operation because there are no peritoneal adhesions. During reoperation, cysto-biliary fistulas were observed at the site of endocystectomy in all patients and were closed with sutures. Bile leakage detected later on was treated with interventional therapies such as ERC and/or CT-guided drainage. Patients with early bile leakage who underwent reoperation had a significantly shorter hospital stay than patients with late bile leakage. This might be because bile leakages after endocystectomy are often due to cysto-biliary fistulas that are missed or mismanaged during the operation. Since these fistulas are well established before the operation, a long time is needed for a spontaneous healing when treated conservatively. In patients who underwent early reoperation, the problem was immediately solved by closing the biliary fistulas, an all these patients were discharged after a couple of days. Even though both strategies were successful in treating the bile leakage, surgery was a significantly faster solution.

It is known that a longer hospital stay can increase hospital-acquired patient complications and lower patient satisfaction [29,30]. A shorter hospital stay increases bed capacity and patient overflow, thereby increasing the operation capacity. This reduces costs for patients and healthcare systems, while increasing the hospital profit through more efficient bed management. In our study, DRG reimbursements were higher in patients undergoing reoperation (€20,851 vs. €14,078) or ($22,936 vs. $15,485) while the overall costs were lower (€12,881 vs. €16,661) or ($14,169 vs. $18,327). This led to a significantly better cost efficiency for these patients (€2072 vs. €-2097) or ($2,279 vs. $-2,306). Taken together, these results show that early reoperation might benefit patients, the hospital, and care givers, and reduces the use of hospital and personal resources.

The routine use of surgical drains in liver surgery is still under debate [31–33]. Generally, drains are used to identify and treat any leakage or fluid collection. We routinely fit [11] intraoperative drains in the resection site and, in this study, surgical drains identified bile leakages in nine out of 11 patients. This shows that drains might help diagnose bile leakage early on after endocystectomy, thereby allowing an early reoperation. Therefore, we believe that patients undergoing endocystectomy for CE benefit from being fitted with a drain after surgery, particularly high-risk patients (i.e., patients with a cyst diameter >8 cm or pre/intraoperative cysto-biliary fistulas). On the other hand, 10 out of 11 patients with bile leakage needed an intervention or reoperation, indicating that the drains we fitted did not have a therapeutic effect. Published studies on the role of surgical drains have not included patients undergoing endocystectomy for CE. Therefore, further studies are needed to evaluate the role of routine surgical drains in these patients.

Data on the direct costs of CE therapy are scarce. To our knowledge, no study has reported the direct cost of endocystectomy for CE. A recent study in Italy reported similar costs to those we report in patients undergoing surgical treatment for CE (mean cost of €11,033; range: €5,874–€23,077) [34]. These costs included different surgical methods (marsupialization, pericyctectomy, and lobectomy), but the cost of different types of operation were not mentioned. Another study conducted in Sardinia [35] reported a mean surgical cost of €8,856 per patient but did not mention the type of operations. Therefore, is not possible to compare costs between different surgical methods in the current literature. There are also no studies that report costs for patients with and without postoperative complications separately, so we cannot draw conclusions on the effect of complications on hospital costs from the literature. In the current study, patients with complications had significantly higher hospital costs. Furthermore, bile leakage significantly increased hospital costs in our cohort. This finding is in line with the results of other studies showing that bile leakage and other complications have a dramatic impact on hospital costs [36,37] in patients undergoing liver surgeries other than endocystectomy.

This study bears some limitations. Even though the data was collected prospectively, the study had a retrospective design. The sample size was not very large because the study was single center, and therefore the frequency of bile leakage after endocystectomy was low. In our center, the standard surgical therapy for cystic echinococcosis is endocytectomy, and over the years, very rare cases underwent liver resections. Therefore, a comparison between these two methods using our data was not possible. Furthermore, choosing between the two different treatment strategies for postoperative bile leakage (conservative treatment versus reoperation) was based on the time of occurrence. For a bias free comparison, randomization of the patients is necessary. Therefore, concrete evidence and reliable conclusions can not be derived from our data considering the superior strategy of dealing with postoperative bile leakage. Nevertheless, these data revealed the need of future prospective randomized studies to compare these strategies to find the ideal solution for post-endocystectomy bile leakage.

In conclusion, bile leakage was the main complication in patients undergoing endocystectomy. The risk factors for bile leakage were cyst diameters larger than 8 cm and cysto-biliary fistulas. In our patients, early operative management of bile leakage reduced costs and the duration of hospital stay compared with conservative treatments. Based on these data, further prospective studies could be designed to compare outcomes following early reoperation versus conservative therapy to treat bile leakage after endocystectomy.

## Supporting information

S1 Data Primary data set used for analysis. (XLSX)
